# NURSING HOME STAFF UNDERSTANDING AND CONFIDENCE IMPLEMENTING MONTESSORI APPROACHES AFTER TRAINING

**DOI:** 10.1093/geroni/igad104.1640

**Published:** 2023-12-21

**Authors:** Michelle Hilgeman, Katherine Kennedy, Sylvia Haigh, Amy Mochel, Teddy Bishop, Kimberly Curyto, Whitney Mills

**Affiliations:** Tuscaloosa VA Medical Center, Tuscaloosa, Alabama, United States; Providence VA Medical Center, Providence, Rhode Island, United States; Providence VAMC, Providence, Rhode Island, United States; Providence VAMC, Providence, Rhode Island, United States; Tuscaloosa VA Medical Center, Tuscaloosa, Alabama, United States; Center for Integrated Healthcare, VA Western NY Healthcare System, Batavia, New York, United States; VA Providence Healthcare System, Providence, Rhode Island, United States

## Abstract

Staff understanding and confidence delivering non-pharmacological interventions are critical for improving outcomes related to behavioral distress in dementia. We conducted a mixed methods evaluation with frontline staff (nursing, recreation therapists, etc.) at 8 VA Community Living Centers enrolled in a stepped-wedge cluster randomized trial of Montessori-based approaches. Training and data collection were conducted remotely and surveys were shortened to reduce participant burden. Normalization Process Theory (NPT, May et al., 2022) guided questions and analysis. Staff completed N=21 qualitative interviews, N=307 surveys (1-month pre-training and during baseline), and N=906 post-training evaluations. Results revealed 83%-87% passed post-training knowledge exams. NPT surveys reflected Montessori was not a normal part of staff work currently (M=5.58 and 6.35 on a 10-point scale); though they believed it would become a normal part of their work in the future (M=7.11 and 7.68). After training, Montessori was rated as more familiar (M=5.15 to M=6.55, t=-3.12, p=.002), and significantly more staff reported that their role included Montessori delivery/oversight X2=13.26(2), p=.001. Content analysis was used to examine qualitative transcripts for the NPT domain Coherence (e.g., comprehension, self-efficacy, internalization). Three themes emerged: 1) ability to describe Montessori principles, 2) familiarity with Montessori approach, and 3) willingness to try it. All participants were able to describe Montessori, though some reported conditional self-efficacy/confidence. Representative quotes reflect challenges and successes in understanding and early application of the intervention in VA CLC environments. Considerations for pragmatic trial designs amid fluctuating COVID precautions are also discussed (e.g., nursing shortages, implementation and evaluation discrepancies, and site-level variability).

